# Enhanced Proton Conductivity in Y‐Doped BaZrO_3_ via Strain Engineering

**DOI:** 10.1002/advs.201700467

**Published:** 2017-10-27

**Authors:** Aline Fluri, Aris Marcolongo, Vladimir Roddatis, Alexander Wokaun, Daniele Pergolesi, Nicola Marzari, Thomas Lippert

**Affiliations:** ^1^ Thin Films and Interfaces Group Research with Neutrons and Muons Division Paul Scherrer Institute 5232 Villigen PSI Switzerland; ^2^ Theory and Simulations of Materials (THEOS), and National Centre for Computational Design and Discovery of Novel Materials (MARVEL) École Polytechnique Fédérale de Lausanne Station 12 1015 Lausanne Switzerland; ^3^ Institute of Materials Physics University of Göttingen Friedrich‐Hund‐Platz 1 Göttingen 37077 Germany; ^4^ Department of Chemistry and Applied Biosciences Laboratory of Inorganic Chemistry Vladimir‐Prelog‐Weg 1‐5/10, ETH Zürich 8093 Zürich Switzerland

**Keywords:** proton conductor, simulation, strain, thin film, Y‐doped BaZrO_3_

## Abstract

The effects of stress‐induced lattice distortions (strain) on the conductivity of Y‐doped BaZrO_3_, a high‐temperature proton conductor with key technological applications for sustainable electrochemical energy conversion, are studied. Highly ordered epitaxial thin films are grown in different strain states while monitoring the stress generation and evolution in situ. Enhanced proton conductivity due to lower activation energies is discovered under controlled conditions of tensile strain. In particular, a twofold increased conductivity is measured at 200 °C along a 0.7% tensile strained lattice. This is at variance with conclusions coming from force‐field simulations or the static calculations of diffusion barriers. Here, extensive first‐principles molecular dynamic simulations of proton diffusivity in the proton‐trapping regime are therefore performed and found to agree with the experiments. The simulations highlight that compressive strain confines protons in planes parallel to the substrate, while tensile strain boosts diffusivity in the perpendicular direction, with the net result that the overall conductivity is enhanced. It is indeed the presence of the dopant and the proton‐trapping effect that makes tensile strain favorable for proton conduction.

## 1 Introduction

Over the last decade, considerable efforts have been devoted to studying the effects of strain (lattice distortions) on the oxygen‐ion conductivity in oxides such as yttria‐stabilized zirconia and doped ceria.^1^, ^2^, ^3^, ^4^ The interest arose because tensile strain leads to higher conductivities at reduced temperatures for applications in solid‐oxide fuel and electrolyzer cells (SOFCs/SOECs), sensors, and oxygen pumps.^5^, ^6^, ^7^, ^8^, ^9^ Very few studies extended the investigation of the effect of strain on the ionic conductivity to high‐temperature proton conductors (HTPCs), a class of ionic conductors equally important as oxygen‐ion conductors in applied electrochemistry.^5^, ^6^, ^7^, ^8^, ^9^, ^10^, ^11^, ^12^ HTPCs promise great potentials as SOFCs/SOECs electrolyte membranes. The main advantage with respect to oxygen‐ion conducting oxides is the significantly lower activation energy required for proton migration in solids. This property allows preserving good ionic conductivity at relatively low temperatures. Considering that the high operating temperature is the most important drawback of current SOFC/SOEC technology, the full exploitation of HTPC materials could represent a major breakthrough for sustainable and renewable electrochemical energy conversion.^9^ In addition, SOFCs (SOECs) based on proton‐conducting oxides as the electrolyte produce water as by‐product at the cathode (anode) side. This avoids the dilution of the fuel in SOFCs and the need of H_2_ separation membranes in SOECs used for steam electrolysis.^13^

Due to its chemical stability and high bulk proton conductivity in the low temperature range (300–500 °C), the perovskite oxide Y‐doped BaZrO_3_ (BZY) is probably the most representative proton‐conducting oxide.^9^, ^12^ The partial Y^3+^ substitution into the Zr^4+^ sites creates the oxygen vacancies required for proton uptake through dissociative water vapor absorption.^11^ The proton migration occurs through a Grotthuss‐type mechanism and the ionic conductivity can be described as

(1) $$\sigma_{\mathrm{ion}}=\frac{\sigma_{0}}{T}\;e^{-E_{A}/k_{B}T}$$

where *E*_A_ is an effective activation energy, “effective” because for single jumps different energy barriers can be present depending on the local surrounding environment. The preexponential factor σ_0_ is proportional to the density of charge carriers, to the attempt frequency for hopping, and to the distance between hopping sites.^14^ For BZY the maximal conductivity is achieved for dopant contents between 15% and 20%, with a typical bulk *E*_A_ ≈ 0.45 eV.^11^, ^15^, ^16^, ^17^

Theory and experiment suggest that the aliovalent dopant required for proton uptake creates a negatively charged lattice site, thus becoming an inherent trapping center for protons. Therefore, the proton‐dopant association energy largely contributes to the total effective *E*_A_, which has been suggested to be as low as about 0.15 eV in an ideal trap‐free BaZrO_3_ physicochemical environment_._ 18, 19

When an ion conductor is strained, the interatomic spacing changes, modifying the electron density in the crystal, the potential energy landscape, and as a consequence the ionic conductivity σ_ion_.^1^ For oxygen‐ion conductors, it was found that biaxial tensile strain enhances the conductivity by decreasing *E*_A_, while compressive strain leads to the opposite effect.^1^, ^2^, ^3^, ^4^ For proton conductors, only the effect of an isotropic pressure, i.e., a reduced lattice spacing along all three spatial axes, applied to sintered powders of Y‐doped BaZrO_3_ and BaCeO_3_ was investigated.^20^, ^21^, ^22^ The extent of the reported effect varies enormously, however all studies show a larger *E*_A_ (lower σ_ion_) in compressive stress compared to the relaxed structure. If this trend can be extrapolated, a lower *E*_A_ and thus higher σ_ion_ at low temperatures may be obtained under tensile stress. Nevertheless, this has never been experimentally investigated. In contrast, computational studies predicted the opposite effect, namely that the diffusion coefficient (*D* ∝ σ_ion_) of BZY monotonically decreases from compressive to tensile strain under isotropic pressure.^23^, ^24^ Thus, higher conductivities should be found under compressive stress. In the case of biaxial stress, i.e., a lattice distorted along two axes with the third free to adapt, calculations predicted a parabolic trend of *D* as a function of stress, with the maximum diffusivity occurring under compressive stress.^23^, ^24^ The discrepancy between theory and experiment would suggest that the simulation models may have overlooked some fundamental aspects of the conduction mechanism in BZY. However, it should be remarked that the number of experimental studies is very limited and that the magnitude of the effect differs enormously^20^, ^22^ and changes with the synthesis method.^21^

To further improve our understanding of the proton migration in solids it is of utmost importance to clarify the effect of strain by (a) fabricating HTPCs with well‐defined strain state, (b) extending the experimental investigation to tensile strain to identify what strain state leads to the maximum conductivity, (c) quantifying how much strain can affect the charge transport, and (d) developing a simulation model capable to rationalize the experimental findings.

Here, the relation between proton conductivity and strain is investigated using highly ordered epitaxial thin films with a nominal composition of BaZr_0.8_Y_0.2_O_3_ (20BZY) grown by pulsed‐laser deposition in different and well‐controlled strain.

## 2 Results and Discussion

### 2.1 Epitaxial Thin Films as Model Systems for Single Crystals

The 20BZY films are grown on insulating substrates in order to measure their conductivity in‐plane (parallel to the substrate surface). The in‐plane strain is controlled by tuning the film‐to‐substrate lattice mismatch

(2) $$f=\left(a_{\mathrm{Substrate}}^{0}-a_{\mathrm{Film}}^{0}\right)/a_{\mathrm{Film}}^{0}$$

where *a* ^0^ indicates the relaxed lattice constant. Under ideal epitaxy conditions, typically realized for small thickness (few nm) and *f* < 1%,^25^, ^26^ the film adopts the in‐plane lattice constant of the substrate and the lattice mismatch equals the in‐plane strain in the film. As the thin film grows, different crystalline defects (dislocations, grain boundaries, surface roughening) can reduce the strain so that the in‐plane average lattice constant of the film $a_{\mathrm{Film}}\neq a_{\mathrm{Substrate}}^{0}$ and the effective strain becomes $\varepsilon \;=(a_{\mathrm{Film}}-a_{\mathrm{Film}}^{0})/a_{\mathrm{Film}}^{0}$.^25^, ^26^, ^27^, ^28^

We use (001)‐oriented MgO substrates (*a* = 4.212 Å) that provide an excellent platform for growing epitaxial 20BZY (*a* = 4.223 Å) films,^29^, ^30^, ^31^ having the same cubic symmetry and a small lattice mismatch of −0.26% (compressive). Furthermore, MgO is highly insulating, which is a prerequisite for in‐plane electrical characterizations of thin films. To relax the in‐plane compressive strain that an ideal 20BZY epitaxial film would develop on MgO, and to further push the lattice distortions toward tensile strain, we grow a buffer layer of 40% Ce‐doped BaZrO_3_ (BZC) in situ between the substrate and the film. Below 800 °C, BZC has an orthorhombic cell with a pseudocubic lattice parameter of about 4.3 Å,^32^ thus larger than either MgO or 20BZY. BZC is also expected to be a good electrical insulator in the gaseous environment (humidified Ar) and temperature range (<600 °C) where the proton conductivity is typically investigated. To carefully tune the strain, the generation and evolution of the film stress (proportional to the strain over the elastic modulus) was monitored in situ with a multi‐beam optical stress sensor (MOSS) by measuring the change in substrate curvature (see Figure S1 in the Supporting Information).

**Figure 1:**
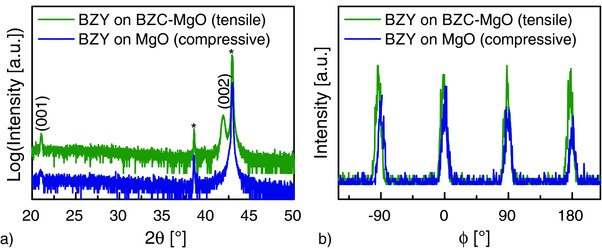
Out‐of‐plane ω/2θ scans a) and φ scan of the (103) reflection to probe in‐plane orientation b). Examples of a BZY film on MgO and a tensile strained BZY film on a BZC film are shown. * labels the substrate reflections, MgO(002); the weaker results from Cu Kβ. The (001) reflection is forbidden for MgO, but present, though weak for BZY and BZC. The (002) reflection of BZC is clearly visible, while the (002) reflection of BZY coincides with the substrate reflection.

For all films, X‐ray diffraction (XRD) confirmed the expected epitaxial in‐ and out‐of‐plane orientations (**Figure** **1**). Only the (002) reflection of substrate and film and the (001) reflection of the film, which is forbidden for the rock salt structure of the substrate, are visible in the out‐of‐plane measurement (Figure 1a). Ce‐doped BaZrO_3_ (BZC) has a larger lattice constant than BZY, therefore the BZC(002) reflection is clearly visible while the BZY(002) reflection is only discernible as a shoulder of the MgO(002) substrate reflection. The asymmetric (103) reflection of the film (forbidden for the substrate) is found at intervals of 90° showing the cubic in‐plane symmetry, aligned with the substrate lattice (Figure 1b).

The chemical composition of the films was analyzed with Rutherford backscattering and particle induced X‐ray emission and found to be Ba_0.98_Zr_0.82_Y_0.21_O_2.85_ (see Figure S2 in the Supporting Information); hereafter, this composition will be referred to as BZY. It is worth highlighting that such a relatively small Ba deficiency, which is often reported in literature for barium zirconate samples,^33^ does not significantly affect the conducting properties of the material and the expected value of bulk activation energy of about 0.45 eV is typically measured.^16^

**Figure 2:**
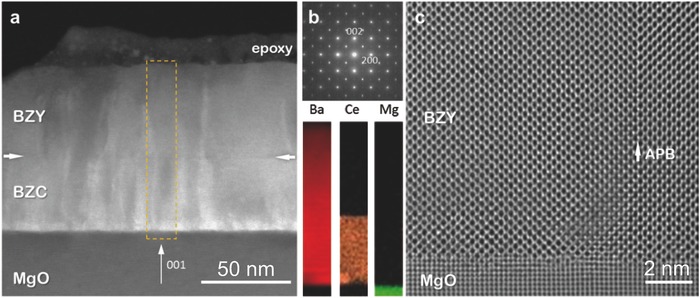
High‐resolution transmission electron microscopy. a) High angle annular dark field scanning transmission electron microscopy image of a BZY/BZC bilayer on MgO(001). b) Selected area electron diffraction pattern and energy electron loss spectroscopy maps. c) High‐resolution transmission electron microscopy image of BZY/MgO interface showing an antiphase boundary defect.

High‐resolution (scanning‐)transmission‐electron microscopy and electron‐energy‐loss spectroscopy were used to study the local structure and composition (**Figure** **2**). All films showed a cube‐on‐cube epitaxial growth, with columnar morphology (Figure 2a), where continuous lines of low‐angle grain boundaries originating from the BZC/MgO interface propagate across the BZY/BZC interface. Electron diffraction (Figure 2b) did not reveal the presence of secondary phases or strongly tilted grains, and the analysis of the chemical profile demonstrated the absence of chemical inter‐diffusion at both interfaces. As an example, Figure 2c shows a sharp BZY/MgO interface and a typical defect (vertical antiphase boundary). The high crystalline quality of the films allows their use as a model system for single crystalline bulk.

A complete structural, morphological, and compositional characterization of proton‐conducting thin films as presented here is rarely reported in literature. For this work, this is of particular importance since it allows us to exclude side effects on the conducting properties.

### 2.2 Strain Engineering Through In Situ Stress Monitoring

When a strained film grows, the substrate bends to minimize the total elastic energy of film and substrate. The resulting curvature is proportional to the stress–thickness product, as described by Stoney's equation.^27^, ^34^, ^35^ A positive (negative) sign for the curvature indicates tensile (compressive) stress in the growing film. In the case of an almost coherent hetero‐interface the stress that forces the substrate to bend arises from the different lattice parameters of the two materials adapting to one another at the interface. The relative change of the radius of curvature of the substrate depends on its elastic properties and can typically be in the range of several kilometers. Nevertheless, even when coupling two relatively hard oxide materials as substrate and film, the experimental setup is sensitive enough to detect relative changes of curvature induced by the deposition of films less than 2 nm thick. With increasing film thickness, the substrate continues to bend as long as more elastic energy can be stored in the system. Above a critical thickness, the total elastic energy of the film becomes so large that it is energetically favorable to introduce crystallographic defects to release the strain.^25^, ^26^, ^36^

**Figure 3:**
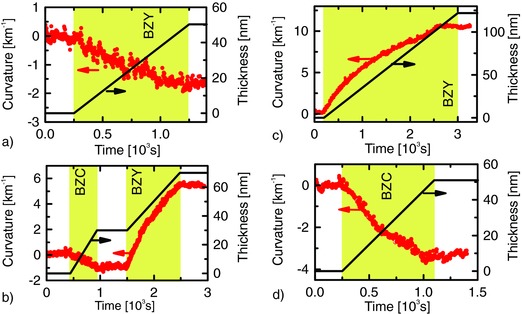
Examples of in situ curvature measurements. The evolution of the curvature with time before, during and after the film growth on MgO is shown in a) for BZY and in b) for the BZY on BZC. The growth of a ≈120 nm thick BZY layer on BZC is shown in c) and d) shows the growth of a ≈50 nm thick BZC film on MgO.

During the growth of BZY films on MgO the MOSS shows the development of an in‐plane compressive stress of the films (**Figure** **3**a), consistent with the lattice mismatch. As expected, also the BZC films on MgO grow with an in‐plane compressive stress but during the successive growth of BZY on BZC‐buffered MgO, the substrate bends in the opposite direction indicating in‐plane tensile stress (Figure 3b). Figure 3c shows, as an example, the curvature evolution during the growth of an in‐plane tensile strained BZY film on BZC‐buffered MgO (see Figure S1 in the Supporting Information and corresponding remarks). Figure 3b,c demonstrates that thin BZY films with different strains can be fabricated by selecting different stages of relaxation corresponding to different thicknesses. That way, however, a strain‐dependent conductivity is not distinguishable from a thickness‐dependent conductivity arising from interface or surface effects, where the film may have different conductive properties (e.g., due to a compositional gradient). For this work, we engineered a set of samples where strain and thickness do not correlate by selecting different stages of relaxation of the buffer layer. To quantify the strain, reciprocal space mapping (RSM) is used.

Based on MOSS and RSM measurements, two thicknesses of 15 and 30 nm were selected for the BZC buffer layer in order to provide a lattice mismatch with the relaxed BZY structure of about 0.3% and 0.9%, respectively.

On the 15 nm BZC buffer layer, BZY films of 15, 22, and 87 nm were grown. The first shows in‐plane strain ε ≈ 0.2%, while films of a thickness greater than ≈20 nm show a relaxed structure. On the 30 nm buffer layer, BZY films were grown with thicknesses of 15, 22, and 43 nm, all showing an in‐plane strain ε ≈ 0.7%. The earlier relaxation onset of BZY on the thinner buffer layer could be due to more pronounced surface roughness during the early‐growth stage, whereas a better interfacial smoothness may be achieved during the further growth of the BZC layer.

Samples under compressive strain are added to the above described set of tensile strained or relaxed samples by growing BZY films on MgO. These films show a strain ε ≈ −0.3% indicating that the lattice mismatch of the two materials is preserved. In general, the BZY films grown on the buffer layers showed a residual in‐plane strain smaller than their respective lattice mismatches. The more favorable stress relaxation for films grown on buffer layers is probably due to the crystalline defects described before (Figure 2).

**Figure 4:**
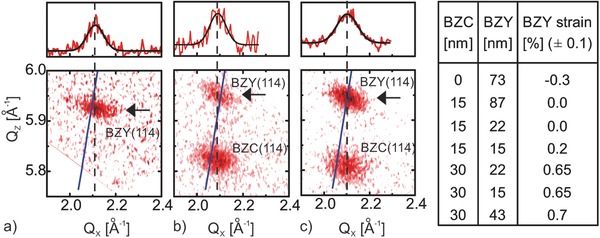
Reciprocal space mapping for strain characterization. Examples for a BZY film on MgO a), a tensile strained BZY film on a 30 nm BZC film b) and a relaxed BZY film on a 15 nm BZC film c) are shown. The two buffer layers provide two growth platforms with different in‐plane lattice constants of 4.23 and 4.26 Å for the 15 and 30 nm BZC layers, respectively. The solid line indicates where in‐ and out‐of‐plane lattice constants are equal. The origins of the line profiles (Δ*Q*_Z_ = 0.01 Å^−1^) shown in the top part are indicated with arrows. The dashed line indicates the center of the reflection as determined from fitting the line profile. Based on the error of fitting the line profiles, the error in strain is about ±0.1%. The BZY strain values measured by RSM for all samples are listed.

**Figure** **4** shows the RSMs for 22 nm BZY films grown either on MgO or on the 15 and 30 nm BZC buffer layers. The first film is 0.3% compressively strained in‐plane, the latter is under 0.65% in‐plane tensile strain, while the film grown on the 15 nm BZC buffer layer is fully relaxed. The strain values and thicknesses of all samples used for the electrical characterizations are summarized in Figure 4.

### 2.3 Tensile Strain Reduces *E*_A_ for Proton Conduction

**Figure 5:**
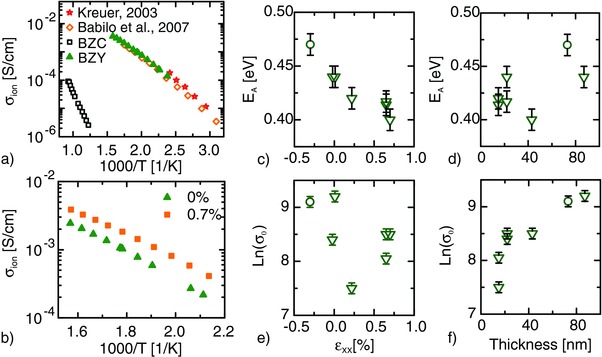
The proton conductivity was measured in humidified Ar. A relaxed BZY film on BZC is compared to the grain interior conductivity reported in literature^11^, ^43^ and to the conductivity of the BZC buffer layer a). In (b) the conductivity of two 22 nm thick films are compared. The different buffer layer thicknesses 15 and 30 nm induced different tensile strain values in BZY, namely 0% and 0.7%, respectively. In c–f), the triangles represent tensile strained BZY films on BZC buffer layers while the circle represents the compressive strained BZY film on MgO. Correlations between the *E*_A_ and the strain c) as well as between Ln(σ_0_) and the thickness f) are found. In contrast, there is no correlation of *E*_A_ and thickness d) or Ln(σ_0_) and strain e). The indicated error bars result from the fit of the linearized Arrhenius equation. The error for the strain is estimated as ±0.1% (Figure 3), corresponding about to the width of the symbols. Examples of complex impedance plane plots are shown in Figure S3 (Supporting Information).

The proton conductivity of the samples listed in Figure 4 is measured by impedance spectroscopy in humidified Ar atmosphere. The conductivity of strain‐free films agrees well with that of the 20BZY grain interior, as reported in literature^11^, ^37^ (**Figure** **5**a). Figure S3a in the Supporting Information shows the temperature dependence of the corresponding complex impedance plane plots.

As expected, BZC shows a very low conductivity compared to BZY making its contribution to the total conductivity negligible. The BZC conductivity is about five orders of magnitude lower than BZY at 350 °C, and the difference increases at lower temperatures due to the higher activation energy (Figure 5a).

Deuteron conductivity is measured to confirm that protons are the main charge carriers under the selected experimental conditions via the isotope effect.^38^, ^39^ Assuming a harmonic oscillator, the attempt frequency (proportional to the preexponential factor σ_0_ of Equation (1)) would be lower by a factor of $\sqrt{2}$ for the two times heavier deuterons as compared to protons. Taking quantum mechanics into account, the lowest energy level in a potential well is lower (corresponding to higher activation energy) for a deuteron than for a proton. This is reflected in different activation energies for protons and deuterons. Here, σ_0_ is higher for deuterons, by a factor of 1.2 ± 0.2 and the activation energy is higher by 0.04 ± 0.01 eV (Figure S4, Supporting Information). This is in agreement with values reported for 10BZY^40^ and other proton‐conducting oxides.^38^, ^39^ In conclusion, the isotope effect is clearly observed, confirming the protonic nature of the charge carriers. It is interesting that in different proton conductors, the ratio between the σ_0_ is often smaller than $\sqrt{2}$, which likely reflects that the vibration of the oxygen sublattice is more important for proton transfer than the vibrations in the O—H bond.^20^

Further, the conductivity in dry O_2_ was measured. In this case, the dominant charge carriers should be electron holes and the activation energy should be higher.^41^ This is confirmed with our samples that show *E*_A_ = 0.78 ± 0.01 eV in dry O_2_.

Figure 5b shows the conductivities of two BZY films with the same thickness of about 22 nm but different strain: 0 and 0.7% in‐plane tensile. In Figure S3b of the Supporting Information, the corresponding complex impedance plane plots acquired at about 325 °C are shown. The conductivity clearly increases under tensile strain and is about twice the value of the relaxed structure around 200 °C. To our knowledge, this is the first experimental evidence of the effects of tensile strain on the proton conductivity in oxides.

Figure 5c shows the effect of strain on *E*_A_ for proton migration in BZY films for strains going from −0.3 to 0.7%. An effective *E*_A_ of about 0.44 eV is obtained for the case of the relaxed structure. The *E*_A_ increases to 0.47 eV for compressive strain, while it decreases to 0.42–0.39 eV with increasing tensile strain. Figure 5d clearly shows that *E*_A_ does not correlate with the thickness of the BZY film. Fitting the data to the linearized Equation (1) shows that Ln(σ_0_) does not depend on strain (Figure 5e) while it decreases for thicknesses < 50 nm when reducing the film thickness (Figure 5f). Since σ_0_ is proportional to the density of mobile charge carriers, this finding suggests the presence of an interface and/or surface layer a few nanometers thick that does not contribute to the conduction. In fact, the presence of a 3–4 nm thick, proton‐rich layer with altered composition and low proton mobility has been recently reported for In‐doped BaZrO_3_ thin films.^42^ Also, theoretical simulations predicted the formation of a sub‐surface layer with low proton mobility in BZY.^43^ It is important to highlight that the thickness dependence of σ_0_ for small thicknesses does not affect the conclusions discussed above about the effect of strain on the activation energy whose value does not correlate with the film thickness.

The measured effect of strain on *E*_A_ is qualitatively consistent with an extrapolation of the experimental data of hydrostatically compressed powders^20^, ^21^ to tensile strain, while contradicting theoretical predictions.^23^, ^24^ This suggests that the way proton conduction is treated in theoretical simulations needs to be reinvestigated.

### 2.4 The Importance of Considering Proton Trapping and Isotropic Diffusion

We performed several first‐principles molecular dynamic (FPMD) simulations of the diffusion coefficient, following an activated process with a strain‐dependent activation energy: $D(\varepsilon ,T)=D_{0}e^{-E_{A}(\varepsilon )/kT}$, as suggested by our experiments.

Due to dynamical effects, it can be difficult to identify a reaction coordinate for the proton‐transport process. FPMD circumvents this problem by monitoring, under equilibrium conditions, the temperature dependence of the diffusion coefficient, which is supposed to be activated by the same microscopic processes as the experimentally measured protonic conductivity σ_ion_. This methodology inherently accounts for proton trapping, bond breakings and strain effects, even if reaching size and time convergence in FPMD simulations is often a challenging task. In order to reduce computational cost, the optimal strain direction is identified by the condition $\frac{\partial E_{A}}{\partial \varepsilon }=0$. Since our experiments show a strain independent preexponential factor, this condition is rewritten as $\frac{\partial D}{\partial \varepsilon }$ = 0. Therefore, only diffusion coefficients are needed to identify the optimal strain direction, activation energies being much more computationally expensive. Finally, two model systems, either doped or undoped, are used to simulate the different local environments explored by a diffusing proton. The doped system consists of 41‐atom supercell alternating YO_6_ and ZrO_6_ octahedra, while the undoped system of the same sample size contains only ZrO_6_ octahedra. While preexponential factors are certainly affected by the choice of the positions of the octahedra containing the dopant into the simulation cell, the activation energies are determined by microscopic processes involving YO_6_ and ZrO_6_ inter and intraoctahedral diffusion. The doped simulation cell has been devised in order to enhance this kind of transport mechanism and favor the statistical sampling. We stress that in our experiments both environments, doped and undoped, are present simultaneously. Nevertheless, simulating the entire system via FPMD would be computational demanding, requiring much bigger simulation cells. With our approach we aim at analyzing separately the different diffusion processes, without losing information about chemical and dynamical effects. The motion of one single carrier for at least 500 ps has then been monitored. Strain effects are tackled performing all simulations at five different representative strain levels: no strain, ±1.5%, and ±3%.

**Figure 6:**
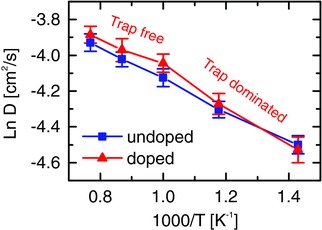
Isotropic diffusion coefficients for the undoped and doped systems. In the latter case we observe a change of activation energy due to the transition from a trap‐dominated to a trap‐free regime. The statistical significance of the difference between the doped and undoped system is discussed in Figure S5 (Supporting Information).

First, we consider unstrained lattices for validation purposes. For the undoped system, i.e., without proton‐trapping effect, our simulations predict *E*_A_ = (0.18 ± 0.02) eV, in agreement with previous simulations^19^, ^44^ and with the experimental *E*_A_ for trap‐free migration.^18^ For the doped system, however, a bending in the Arrhenius plot is observed (**Figure** **6**). This is due to the presence of proton trapping which gradually increases the effective *E*_A_ below 700 °C. At higher temperatures, the trap‐free behavior is recovered since the kinetic energy overtakes the proton‐dopant association energy.^18^ The characteristic stretch and wag frequencies of proton motion are well reproduced by the simulation of the unstrained system as well (see Figure S6 in the Supporting Information).

**Figure 7:**
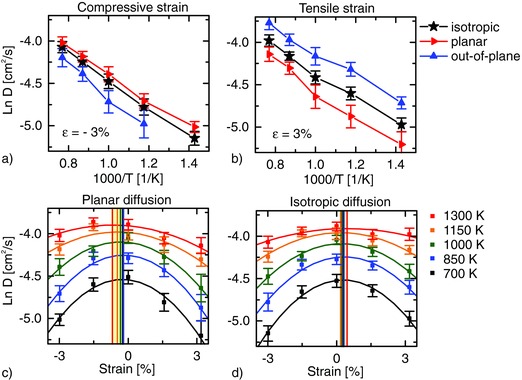
FPMD simulation of the diffusion coefficients for the doped system. The dependence of the diffusion coefficient on the strain is shown at different temperatures for a) planar and b) isotropic diffusion for the doped system. Vertical lines indicate maxima fitted for every temperature. The spread of these independent measurements shows that the optimal strain direction can be clearly identified. For −3% compressive c) and for 3% tensile biaxial strain d), the isotropic, the planar and the out‐of‐plane diffusion coefficients are reported. The equivalent of this figure for the undoped system is in Figure S7 (Supporting Information).

The different qualitative dynamics characterizing proton diffusion in the strained materials is clarified decomposing the diffusion coefficient into a planar (along planes parallel to the strain) and an out‐of‐plane diffusivity. Clearly, as reported in **Figure** **7**a,b for representative ±3% strain levels, the out‐of‐plane diffusivity dominates for tensile strain, whereas a strong 2D confinement of the charge transport under compressive strain is observed. This effect is observed both in the doped and undoped systems (see Figure S7 in the Supporting Information).

When plotting the diffusion coefficient as a function of strain to identify the optimal strain direction, a qualitatively different behavior is instead observed for the doped and undoped systems. In all cases, the diffusivity under ±1.5% and ±3% strain is lower than that of the relaxed structure and decreases with increasing the strain in both directions. This implies that, at constant temperature, *D*(ε) has a maximum between ±1.5%. This qualitative behavior is reported in literature also for biaxially strained BZY^23^ and yttria‐stabilized zirconia (oxygen‐ion conductor).^45^ Our experimental data may also be in agreement with this behavior. The trend shown in Figure 5c of the *E*_A_ for strain values between −0.3% and 0.7% can be much better fitted with a parabolic curve rather than linear (shown in Figure S8 of the Supporting Information). This suggests the existence of a “fastest strain”^45^ where the *E*_A_(ε) has a minimum (and *D*(ε) a maximum) slightly above 0.7% tensile. However, this observation cannot be considered a complete experimental validation of the computational results since the electrical characterization of samples with larger in‐plane strain would be necessary. Unfortunately, our efforts to fabricate BZY films with larger strain on the BZC buffer layer were unsuccessful due to strain relaxation.

The fit of the calculated diffusivities (Figure 7c,d; Figure S7c,d, Supporting Information) indicates that at all simulated temperatures the maxima of *D* are situated in the region of tensile strain only for the case of isotropic diffusion in the doped system.

At this level of accuracy, which already permits to identify the optimal strain direction by comparison with the experiment, no departure from parabolic behavior is observed. Furthermore, our results are stable over a wide temperature range.

As a first observation, we note that the computational results support the experiment only in presence of the dopant suggesting that the measured effect of strain is related to the proton‐trapping effect.

Moreover, we note that the calculation of the planar diffusion coefficient leads to a different position (compared to the experiments) of the maximum diffusivity and of the optimal strain (Figure 7c; Figure S7c, Supporting Information). If the transport processes were strictly 2D, the activation energies and a fortiori the optimal strains for the two diffusion coefficients would agree. This not being the case, we conclude that in the doped system, i.e., when the conduction mechanism is largely ruled by proton trapping, the preferred escape direction from the proton trap involves out‐of‐plane charge transfers, consistently with the observed boost of diffusion along that direction under tensile strain (Figure 7a,b). This strong coupling between out‐of‐plane and in‐plane diffusions makes the isotropic diffusion coefficient, rather than its planar counterpart, better suited to describe the experimental findings.

**Figure 8:**
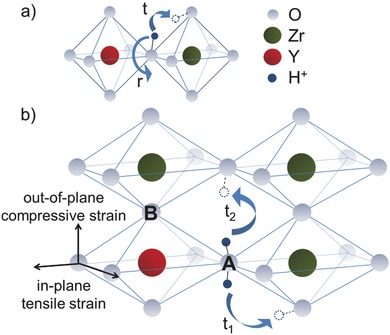
Mechanistic model. a) Rotational diffusion *r* and intraoctahedra proton transfer *t* in the relaxed crystal structure in the trap‐dominated regime. b) Along the plane of an applied biaxial tensile stress the Y–O distance of four oxygen ions (for example in the lattice position A) of the YO_6_ octahedron increases thus lowering the proton‐dopant association energy. The proton escapes the trap through intraoctahedra (t_1_) or interoctahedra (t_2_) out‐of‐plane transfer into adjacent ZrO_6_ octahedra along the direction of a compressive stress. Along the direction perpendicular to the biaxial strain, in the YO_6_ octahedra two oxygen ions (lattice positions B) move closer to the dopant site. These are expected to be trap sites with larger proton‐dopant association energy. Overall, the proton‐dopant association energy in a biaxially strained YO_6_ octahedron is smaller than in the relaxed structure.

Our simulations suggest the following mechanistic model schematically described in **Figure** **8**: In the trap‐free regime, (undoped model system) compressive strain favors the proton migration by shortening the oxygen–oxygen distances thus facilitating the proton transfer between adjacent oxygen ions, as suggested in literature^11^, ^24^ and by the position of all maxima of diffusion computed under these conditions. On the other hand, in the trap‐dominated regime, that is in the presence of the dopant, it is a relatively small tensile strain that enhances the proton migration as the net result of competing mechanisms: Along the direction of the biaxial strain the larger oxygen–oxygen distances hinder the proton transfer but the larger oxygen‐dopant distances of four oxygen ions in the YO_6_ octahedron reduce the proton‐dopant association making it easier for the proton to escape the trap (lattice sites A in Figure 8b).

The enhanced out‐of‐plane diffusivity calculated in tensile strain (Figure 7b) suggests that the most favorable way to escape the trap is via out‐of‐plane intra‐ and interoctahedra hopping (t_1_ and t_2_ in Figure 8b) to adjacent ZrO_6_ octahedra where the out‐of‐plane diffusivity is enhanced in this local trap‐free environment due to the shorter lattice distances in this direction. Along the direction of the out‐of‐plane compressive strain two oxygen ions are closer to the dopant (lattice positions B in Figure 8b) resulting in trap sites with an expected larger proton‐dopant association energy. The net result is an overall smaller proton‐dopant association energy in biaxially tensile strained YO_6_ octahedra.

As far as the in‐plane electrical characterization is concerned, it is irrelevant whether the proton jumps between oxygen ions along planar or out‐of‐plane zigzag pathways. Hence, a fast out‐of‐plane diffusion can indeed boost the conductivity measured in‐plane.

## 3 Conclusion

This study reports the fabrication and characterization of Y‐doped BaZrO_3_ highly ordered epitaxial thin films used as model systems to investigate the effect of strain on the bulk conductivity of acceptor doped perovskite high‐temperature proton conductors. The samples are grown on MgO single crystals using buffer layers of Ce‐doped BaZrO_3_ deposited in situ. Well‐defined strain states can be carefully tuned by selecting different thicknesses of the buffer layer. The evolution of the stress is monitored in situ by multibeam optical stress sensor (MOSS) and the results agree very well with the ex situ measurements of the strain by X‐ray diffraction.

We discovered that tensile lattice strain boosts the proton conductivity along the plane of the applied biaxial stress. In particular, strain can tune the effective activation energy *E*_A_ for bulk proton migration, whose value decreases by ≈0.07 eV when changing the strain from −0.3% (compressive) to 0.7% (tensile). In comparison, a recent study on oxygen‐ion transport in doped ceria shows that a tensile strain of 0.35% lowers *E*_A_ by about 0.05 eV.^4^ Here we show that the effect of strain on the proton conduction in BZY is smaller, yet certainly not negligible, considering that around 200 °C a 0.7% tensile strain doubles the conductivity of the relaxed structure.

Driven by the experimental findings an innovative approach is used for the theoretical simulation of the planar (along the direction of strain), out‐of‐plane, and isotropic diffusion coefficients for the doped and undoped compounds, at different temperatures, and under different strain conditions. Following this approach, a microscopic explanation is proposed where, along the plane of the biaxial tensile strain, two competing mechanisms affect the Grotthuss‐type proton migration between adjacent oxygen ions: The larger distance between the oxygen ions hinders the proton transfer but in the YO_6_ octahedra the larger distance between the oxygen ion and the dopant lowers the proton‐dopant association energy making it easier for the proton to escape the trap at the dopant site. Both, theory and experiment suggest the presence of an optimal tensile strain value that maximizes the conductivity as the net result of these two opposing mechanisms. Within such a frame, the proton migration is described as a three‐dimensional process largely ruled by proton trapping at the dopant and the in‐plane conductivity is enhanced under tensile stress thanks to more favored hopping processes involving across‐plane diffusion from the YO_6_ toward the adjacent ZrO_6_ octahedra.

## 4 Experimental Section

For the thin film growth, an ultrahigh vacuum pulsed laser deposition chamber from Twente Solid State Technology equipped with an MOSS from k‐space Associates was used. The MOSS allows monitoring in situ the change of curvature of the substrate caused by the stress‐induced lattice distortions at the film‐to‐substrate interface. Details of the working principle of this optical wafer curvature measurements are reported in Figure S1 (Supporting Information).

Thin films were fabricated by ablating sintered pellets of 20% Y‐doped BaZrO_3_ and 40% Ce‐doped BaZrO_3_ with a 248 nm KrF excimer laser (Lambda Physics). The laser spot size on the target was 1.5 mm^2^ and a fluence of 2.8 J cm^−2^ and a repetition rate of 4 Hz were used. The MgO substrates (Crystec GmBH) were heated with a radiative heater and the deposition temperature of ≈750 °C was read out with a pyrometer. To monitor the changes of the substrate curvature during the growth the thermal contact between substrate and heating stage could not be provided by metal paste nor any mechanical constrains could be used. To ensure a correct temperature reading with the pyrometer, the unpolished side of the substrates was coated by sputtering with a few hundred nanometer thick Pt film used as thermal absorber. For the pyrometer the emissivity value of platinum black (0.97) was used. A background pressure of O_2_ was set to 1 Pa. The deposition rate was calibrated by X‐ray reflectometry to be 0.013 nm per pulse for BZY and 0.02 nm per pulse for BZC.

X‐ray diffraction was performed with a Siemens D500 diffractometer with Cu Kα radiation (X‐ray reflectometry, ω/2θ and φ scans) and with a Seifert diffractometer with monochromatic Cu Kα1 radiation equipped with a 1D detector. Reciprocal space maps were recorded to quantify the in‐plane lattice constants and strain. The (114) reflection of the BZY and BZC layers was mapped, as this reflection is forbidden for the rock salt structure of the MgO substrate but not for the perovskite structure of the films. The alignment was carried out in reference to the (113) reflection of the substrate. To determine the in‐plane lattice parameter, the fits of the line profiles were performed. From the fit, the in‐plane component *Q*_X_ of the reciprocal lattice vector was determined with an error of about ±0.002 Å^−1^, resulting in an error in strain of about ±0.1%.

Cross‐sectional specimens for transmission electron microscopy (TEM) were prepared with a mechanical polishing followed by Ar^+^ ion milling using a Gatan PIPS 691 with a final milling step of 0.5 keV to reduce surface damage. High‐resolution TEM and high angle annular dark field scanning TEM images were acquired on an FEI Titan 80–300 microscope operated at 300 kV. The microscope was equipped with a corrector of spherical aberration at image side, and a Gatan Quantum 965ER electron energy loss spectrometer.

Electrical characterizations were performed in‐plane by impedance spectroscopy in humidified Ar atmosphere applying an excitation voltage of 1 V in the frequency range between 1 Hz and 1 MHz in the temperature range between 180 and 380 °C. Pt electrodes were deposited on top of the film by sputtering using a 5 nm thick Ti buffer layer to improve adhesion. We used strip‐shaped electrodes patterned by a shadow‐mask (conduction channel of 1 mm length and 4–8 mm width), as well as interdigitated electrodes patterned by UV‐lithography (conduction channel of 0.4 mm length and 31 mm width). Ag paste and Au wires were used to connect the electrodes to the read‐out electronics. A Solartron 1260 gain‐phase analyzer was used together with the software Zplot for the measurement. The software Zview was used to fit the complex impedance plane plots with an RC parallel circuit in the temperature range 210–363 °C. The temperature range was below the start of dehydration but high enough for having values of electrical resistance not so large to significantly affect the accuracy of the measurement.

Car‐Parrinello molecular dynamics,^46^, ^47^ as implemented in the cp.x program of the Quantum‐ESPRESSO^48^ distribution, was used to perform efficient density‐functional theory simulations using the PBE exchange‐correlation functional. Norm‐conserving pseudopotentials from the extensively tested SSSP^49^, ^50^ library were used, with a planewave cutoff of 70 Ryd. The fictitious electronic mass was set to 340 a.m.u., with an integration time step of 8 a.m.u. To avoid an accumulation of numerical errors, eventually causing a departure of the system from the Born‐Oppenheimer surface, five conjugate‐gradient steps were performed every 20 ps, an interval much longer than the typical correlation time associated to proton diffusion in BaZrO_3_. Ionic temperature was controlled by means of a single‐chain Nosé–Hoover thermostat at a frequency of 9 THz, much lower than the characteristic frequencies of protons in this material (see Figure S8 in the Supporting Information). Equilibrium lattice parameters and the Poisson ratio were set using the high‐temperature experimental values^51^ (4.22 Å and 0.237, respectively). Dopant incorporation required the usage of charged cells to reproduce the correct chemistry; for example, in the case of the doped system with chemical formula HBa_8_Zr_4_Y_4_O_24_, where the sum of the nominal oxidation states equals −3, three electrons were added to the simulation cell.

## Conflict of Interest

The authors declare no conflict of interest.

## Supporting information

Supplementary
