# Supplementary material for: Enhanced Proton Conductivity in Y‐Doped BaZrO3 via Strain Engineering
Source: Adv Sci (Weinh). 2017 Oct 27;4(12):1700467. doi: 10.1002/advs.201700467 (PMC5737104; doi:10.1002/advs.201700467)
Supplement: Supplementary file 1 — Supplementary [file ADVS-4-na-s001.pdf]

Copyright WILEY-VCH Verlag GmbH & Co. KGaA, 69469 Weinheim, Germany, 2017.

Supporting Information

for *Adv. Sci.,* DOI: 10.1002/advs.201700467

Enhanced Proton Conductivity in Y-Doped $BaZrO_3$ via Strain Engineering

Aline Fluri, Aris Marcolongo, Vladimir Roddatis, Alexander Wokaun, Daniele Pergolesi, Nicola Marzari, and Thomas Lippert*

Copyright WILEY-VCH Verlag GmbH & Co. KGaA, 69469 Weinheim, Germany, 2016.

Supporting Information

Enhanced Proton Conductivity in Y-doped $BaZrO_3$ *via* Strain Engineering

Aline Fluri, Aris Marcolongo, Vladimir Roddatis, Alexander Wokaun, Daniele Pergolesi Nicola Marzari, Thomas Lippert*

Working Principle of the Multi-beam Optical Stress Sensor (MOSS)

The epitaxial growth of a material with a lattice constant different from that of the substrate induces lattice distortions in the film such that the film adapts the in-plane lattice constant of the substrate. This distortion is quantified as strain, which is proportional to the force per unit area, i.e. the stress, via an elastic constant. The stress corresponds to elastic energy, which can be reduced if the substrate bends. The substrate bends to a curvature at which the reduction in the elastic energy of the film is equal to the energy it takes to bend the substrate. Stoney's equation expresses this relationship between film stress σ_f and substrate curvature $1/\rho$ as

$$\frac{1}{\rho} = \frac{6(1-\nu)}{t_s{}^2 Y} t_f \sigma_f$$

where ν is the Poisson ratio, Y the Young's modulus and t_s and t_f the substrate and film thickness, respectively. The MOSS uses the deflection of parallel laser beams to measure the substrate curvature[1] (Figure S3). The reflected beams are detected by a CCD camera, and their average distance is evaluated by the MOSS software from k-space associates.

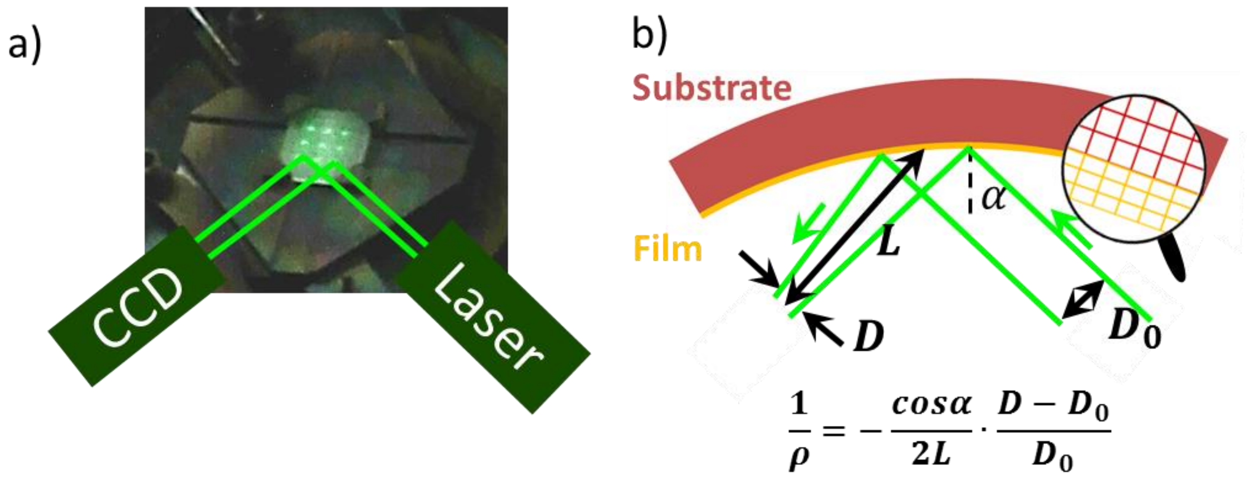

Figure S1. Working Principle of the MOSS. The array of laser spots is visible on the substrate in (a). The sketch in (b) shows how the substrate bends in the case of tensile stress (see magnifying glass) and how that changes the distance D of the reflected laser beams. The substrate curvature 1/ρ is calculated from the angle of incidence α, the distance between sample and CCD camera and the mean differential spacing $(D\text{-}D_0)/D_0$ where D_0 is the distance of the incident beams.

The typically assumed mechanism for strain relaxation in epitaxial thin films is the migration of pre-existing dislocation lines, which creates additional interface dislocations that reduce the average strain. Assuming an equilibrium model (e.g. the Matthews and Blakeslee equilibrium model[2]), the elastic energy of the film remains constant above the critical thickness and, as a consequence, so does the substrate curvature[3-5]. Figure 3c in the manuscript shows the evolution of the curvature during the growth of a BZY film on a BZC-buffered MgO substrate. The total thickness of the film is about 120 nm and the onset of relaxation (i.e. the constant curvature regime) is found at around 85 nm.

Compositional Analysis

Rutherford backscattering (RBS) yields the ratio of Ba : (Zr+Y) : O; the Y and Zr masses are too similar to be separated with this technique. Particle induced X-ray emission (PIXE) is capable of this, and it gives the Y:Zr ratio. Combining the two measurements results in a composition of $Ba_{0.98}Zr_{0.82}Y_{0.21}O_{2.85}$ (BZY) with error in composition for Ba, Y, Zr are +/- 0.02 and for O +/-0.11. The composition is in good agreement with the target composition of 20% Y-doped $BaZrO_3$ (Figure S2).

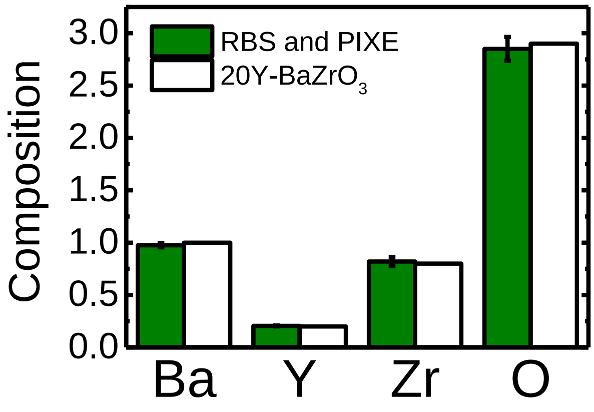

Figure S2: Compositional Analysis of BZY film. The composition is obtained with a combination of RBS and PIXE. For comparison, the composition of 20% Y-doped $BaZrO_3$ is shown.

Additional Remarks on the Electrical Characterisation

The complex impedance plane plots (Figure S3) consist of clean semicircles. For resistances around 20 MΩ and below, a tail corresponding to the polarisation of the electrodes is clearly visible, which hints at the ionic nature of the charge carriers, since the Pt electrodes are blocking ions but not electrons.

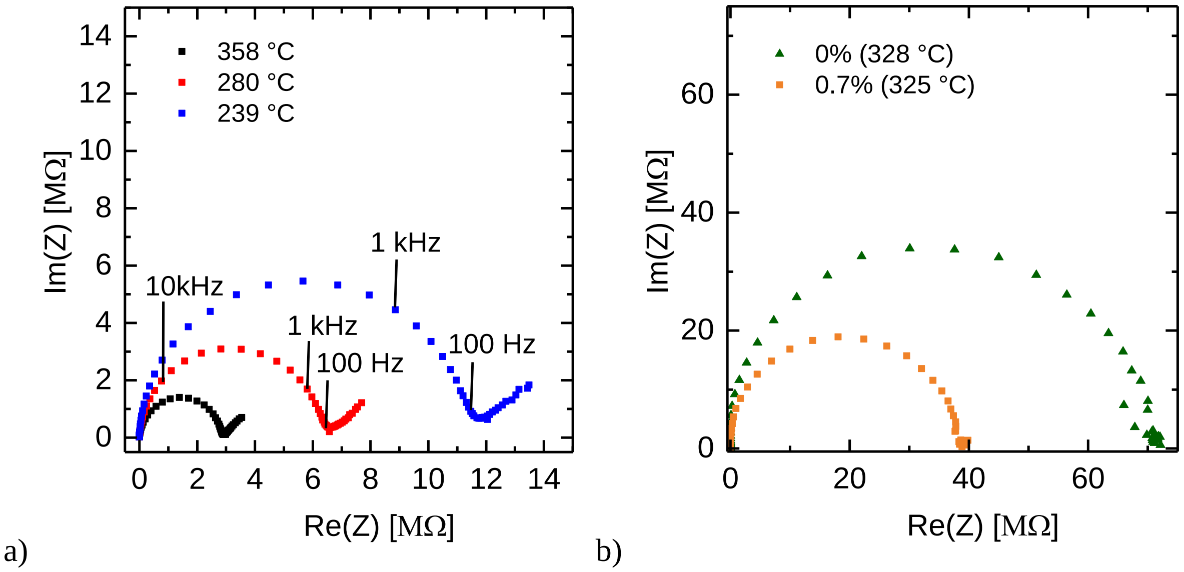

Figure S3. Example of complex impedance plane plots. The plots are shown for three different temperature and selected frequencies are indicated. (a) shows measurements of a fully relaxed BZY film on BZC-buffered MgO. (b) compares two BZY films on BZC-MgO with different strain values but the same thickness and same measurement geometry (same as in Figure 5b in the manuscript).

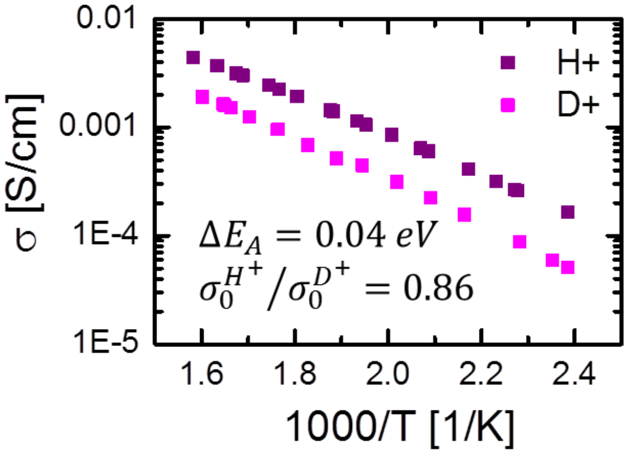

Figure S4. The proton and deuteron conductivity is compared for a BZY film grown on a BZC-buffered MgO substrate.

Additional Remarks on the FPMD Simulations

The in-plane, across plane and isotropic diffusion coefficients are computed according to the definition:

$$D = \lim_{t \to +\infty} \frac{1}{2dt} \sum_{i=1}^{d} \langle [x_i(t) - x_i(0)]^2 \rangle,$$

where d stands for 1, 2 or 3 according to transport dimensionality. Brackets indicate thermal averages, replaced in the calculations by time averages under the hypothesis of ergodicity.

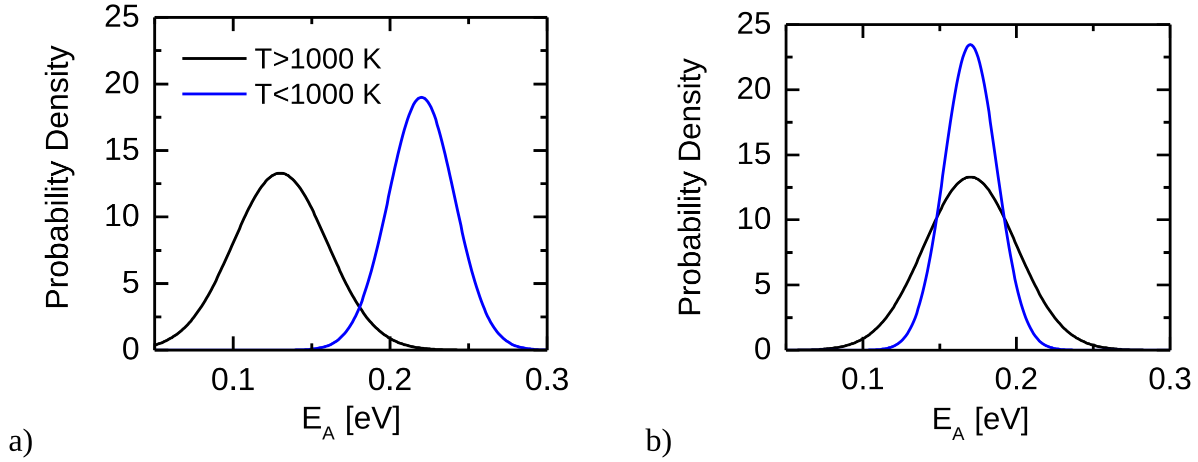

Figure S5. (a) Estimated Gaussian probabilities, in the doped system, for the activation energy in the high and low temperature regimes; (b) refers to the undoped configuration.

Throughout the article 2σ confidence intervals, including the exact values with a probability of approximately 95%, were considered. The error bars offer a visual approach. To quantitatively confirm the change of activation energy in Figure 6 in the manuscript, we fit an activation energy at higher temperatures and observe a result statistically different from the one fitted at lower temperatures. We visualize in Figure S5 the result of such a fitting procedure. Only in the doped system, the estimated Gaussian probabilities do not overlap because of the ongoing transition, as temperature lowers, to a different transport regime.

In Figure S6 a typical Fourier transform of the proton velocity autocorrelation function is reported, with superimposed the frequency of the Nose'-Hoover thermostat, chosen to be distant from the characteristic stretch and wag frequencies[6-7] of proton motion in order to limit possible perturbing effects to proton dynamics. When computing the power spectra of non-diffusing elements (not reported) we observed a dynamical decoupling of the proton motion with respect to the other elements. Physically this implies that to a large extent the contribution of the oxygen matrix to proton diffusion is dominated by the equilibrium Boltzmann distribution of the oxygen-oxygen distances and not by the details of the oxygen dynamics.

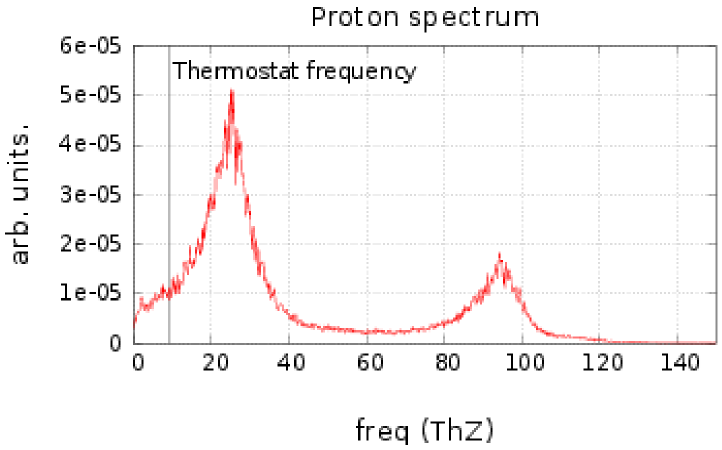

Figure S6. Proton spectrum and thermostat frequency. The plot shows the typical Fourier transform of the proton velocity autocorrelation function, showing the choice of the thermostat frequency with respect to the characteristic stretch and wag frequencies of proton motion.

Figure S7 is the equivalent to Figure 7 in the manuscript for the undoped system. In the case of planar as well as for isotropic diffusion, the maximal diffusivity is found under compressive strain.

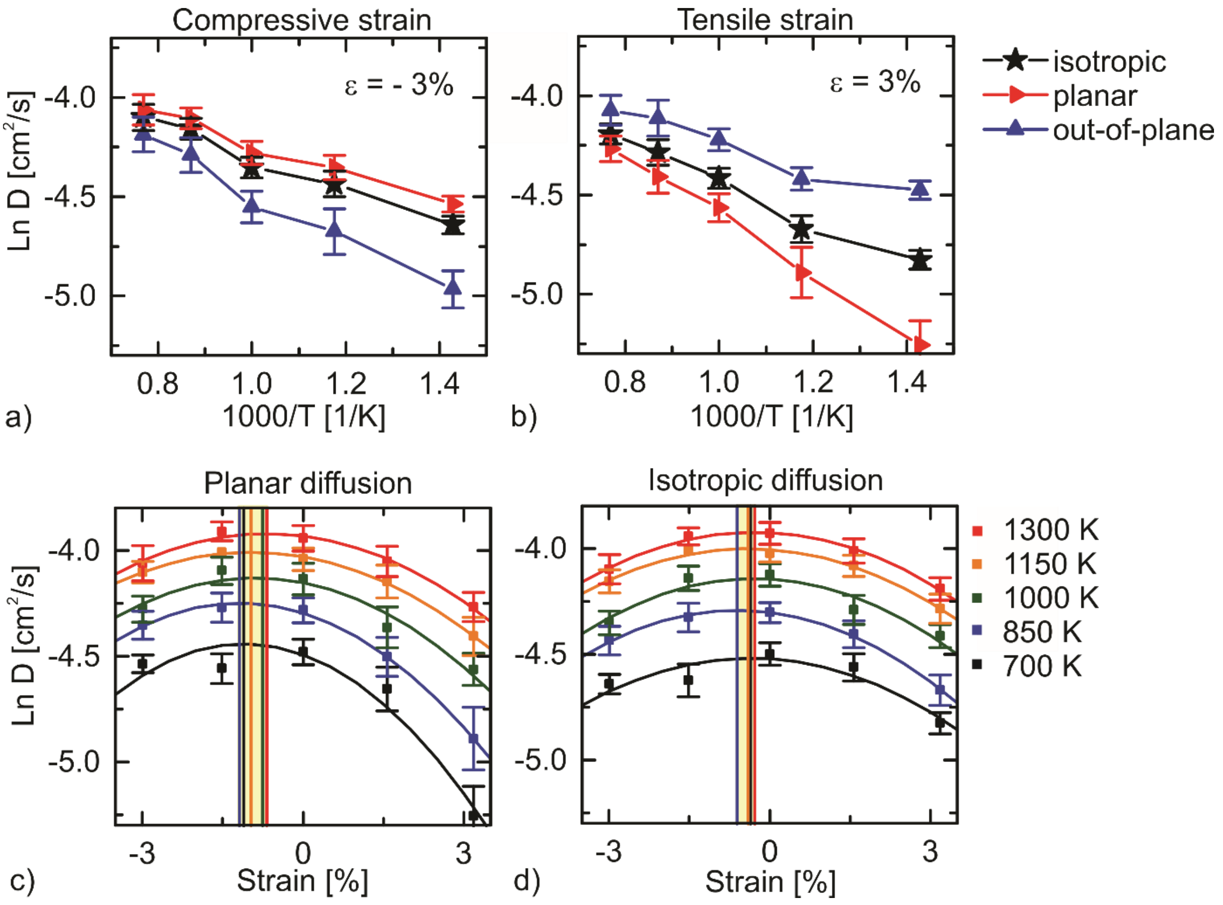

Figure S7. FPMD simulation of the diffusion coefficients for the undoped system. The dependence of the diffusion coefficient on the strain is shown at different temperatures for (a) planar and (b) isotropic diffusion for the doped system. For -3% compressive (c) and for 3% tensile biaxial strain (d), the isotropic, the planar and the out-of-plane diffusion coefficients are reported.

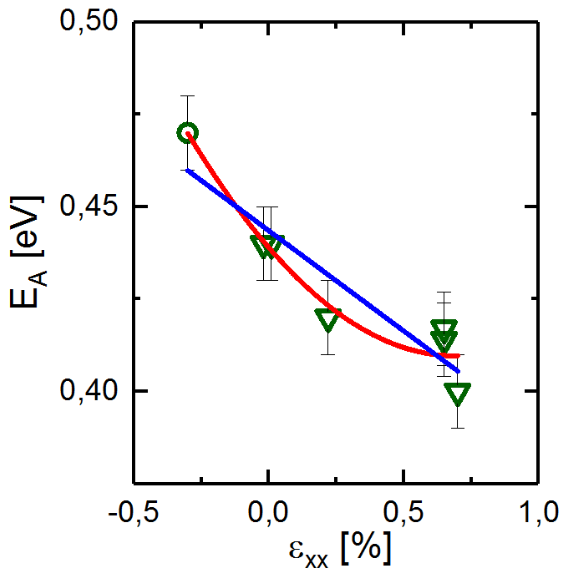

Figure S8. Parabolic vs. linear fit of experimental data. The linear fit (blue) results in a mean square error of 4.09, while the parabolic fit (red) is clearly better, with a mean square error of 1.78.

In Figure S9 we report the behaviour of the fictitious electronic and ionic kinetic energy, showing that the Car-Parrinello molecular dynamics scheme could be efficiently applied.

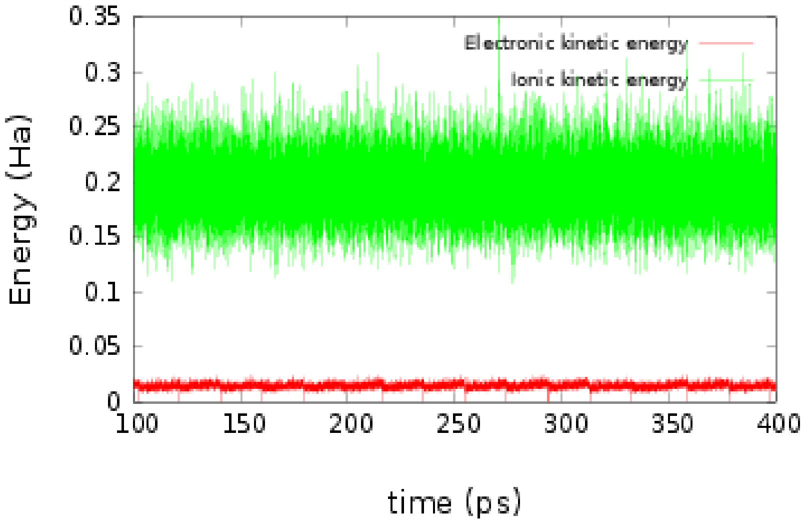

Figure S9. Adiabatic condition for Car-Parrinello molecular dynamics. The plot shows the fictitious electronic and total ionic kinetic energy of the system studied at a representative temperature of 1000K, proving that it is possible to achieve stable, adiabatic dynamics, in

combination with a conjugate gradient restart technique every 20 ps in order to avoid exponential accumulation of numerical errors.

[1] L. B. Freund, S. Suresh, L. B. Freund, S. Suresh., *Film Stress and Substrate Curvature*; Cambridge University Press, 2004.

[2] J. W. Matthews, A. E. Blakeslee, *Journal of Crystal Growth* **1974**, *27*, 118-125.

[3] M. Hanbücken, *Stress and Strain in Epitaxy: Theoretical Concepts, Measurements and Applications*; Elsevier: Amsterdam, 2001.

[4] S. Suresh, L. B. Freund, *Thin Film Materials: Stress, Defect Formation and Surface Evolution*, Reprinted with corr. 2006 ed.; Cambridge University Press: Cambridge, 2006.

[5] J. E. Ayers, *Heteroepitaxy of Semiconductors: Theory, Growth, and Characterization*; CRC Press Taylor & Francis Group: Boca Raton, 2007.

[6] M. E. Björketun, P. G. Sundell, G. Wahnström, *Physical Review B - Condensed Matter and Materials Physics* **2007**, *76*.

[7] M. Fronzi, Y. Tateyama, N. Marzari, M. Nolan, E. Traversa, *Materials for Renewable and Sustainable Energy* **2016**, *5*, 14.
